# Abdominal aortic aneurysm presenting to the orthopedic clinic as posterior hip and low back pain

**DOI:** 10.1002/ccr3.7443

**Published:** 2023-06-19

**Authors:** Aaron Marcel, John Jovan, Karen Myrick

**Affiliations:** ^1^ Frank H. Netter MD School of Medicine at Quinnipiac University North Haven Connecticut USA; ^2^ University of Saint Joseph West Hartford Connecticut USA

**Keywords:** acute medicine, cardiovascular disorders, emergency medicine, orthopedics, vascular surgery

## Abstract

Abdominal aortic aneurysms (AAAs) are typically asymptomatic. When symptomatic, AAA may present as a chief concern of musculoskeletal hip and low back pain. Assessment in the orthopedic clinic should focus on a holistic examination of the patient.

## Graphical abstract

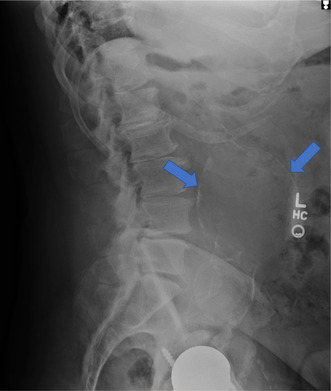

## 1 INTRODUCTION

An abdominal aortic aneurysm (AAA) is a permanent dilatation of the infrarenal aorta that is 3 cm or greater in diameter or equivalent to 1.5 times the normal anteroposterior diameter.^1^, ^2^ Risk factors for AAA include smoking, male sex, age greater than 65 years old, high systolic blood pressure, high body mass index, high serum triglycerides, high low‐density lipoprotein, family history of AAA, coronary artery disease (CAD), atherosclerosis, stroke, and diabetes mellitus with concomitant CAD and peripheral artery disease (PAD).^3^, ^4^, ^5^, ^6^, ^7^, ^8^ Of these, smoking is the greatest contributor to the development of AAA.^3^, ^4^, ^9^ In smokers, growth rate increases by an additional 0.35 mm/year and rupture rate doubles.^7^ Most unruptured AAAs are asymptomatic and are found incidentally while investigating some other pathology.^4^, ^5^, ^7^, ^10^ However, when symptomatic, unruptured AAA may present with unexplained abdominal discomfort and pain that radiates to the back, flank, or groin, as well as a pulsatile abdominal mass with or without a bruit heard at the mass.^4^, ^11^

The generalized pain from a symptomatic AAA may be confused with an orthopedic etiology. A patient presenting with what seems to be a musculoskeletal (MSK) concern may logically lead a clinician toward an MSK differential diagnosis. However, it is important for orthopedic clinicians to consider other etiologies when examining a patient in order to avoid missed diagnoses. In this case report, we discuss a case of an AAA incidentally found during an orthopedic workup for posterior hip and low back pain. Our objectives are (1) to increase awareness of an AAA presenting as orthopedic concerns and (2) to highlight the importance of a thorough history and a holistic physical exam in the orthopedic clinic.

## 2 CASE HISTORY AND EXAMINATION

### 2.1 History of present illness and past medical history

On November 27, 2022, a 70‐year‐old male with a history of bilateral total hip arthroplasty (THA) presented to the orthopedic urgent care clinic with a chief concern of posterior right hip and low back pain persistent over the previous 3 days. The patient noticed the pain after going for a long walk on Thanksgiving Day. The patient denied any recent trauma, injury, or falls. Additionally, he denied abdominal pain and constitutional symptoms such as headache, fever, chills, nausea, lightheadedness, or dizziness. The review of systems was unremarkable.

The patient's past medical history is remarkable for hypertension, hyperlipidemia, CAD, type 2 diabetes mellitus (T2DM), and PAD. Past surgical history includes appendectomy (1972), vasectomy (1981), coronary artery bypass graft (2009), and bilateral THA (right 2014, left 2019). The patient's medications include lisinopril, atorvastatin, and metformin. The patient is allergic to penicillin (hives) and red food dye. He denied any tobacco, alcohol, or illicit drug use.

### 2.2 Clinical examination and findings

The patient presented to the orthopedic urgent care clinic awake, alert, and oriented. He was cooperative with the physical exam and exhibited a normal and appropriate affect. His gait was significantly antalgic. While ambulating, it was evident that he had a painful right leg. Inspection of the right hip and low back revealed focal, reproducible tenderness along the posterior aspect of the right lumbosacral joint. He had no sciatic notch tenderness. There was no significant tenderness to palpation over the midline of the lumbar spine and no significant tenderness over the right flank. Range of motion of the hip was full without any significant discomfort. Neurovascular assessment of both lower extremities was intact.

### 2.3 Differential diagnosis

Given the patient's history and physical exam, causes of both posterior hip and low back pain must be considered.

The differential diagnosis of hip pain is broad and can be compartmentalized into both intra‐articular and extra‐articular causes. Intra‐articular causes include a labral tear, femoroacetabular impingement, and osteoarthritis. Extra‐articular causes can be subdivided by localization of the pain as either anterior, posterior, or lateral. This patient presented with posterior hip pain. Causes of posterior hip pain include sciatic nerve impingement, sacroiliac joint pathology, tendinopathy, muscle strain, and referred pain from lumbar spinal causes.^12^, ^13^ Additionally, this patient has a history of bilateral THA. Therefore, orthopedic complications of THA, such as prothesis wear, aseptic loosening, periprosthetic fracture, and leg length discrepancy, must be considered in the differential diagnosis.^14^

The differential diagnosis for low back pain includes lumbosacral muscle strain, lumbar disc herniation, spondylosis, spinal stenosis, fracture, and malignancy. A muscle strain presents following repetitive or excessive use. On physical exam, pain is typically worse with movement, range of motion is limited, and there is tenderness to palpation of the muscles.^15^ Lumbar disc herniation occurs when an intervertebral disc exerts pressure on a spinal nerve root causing pain and radiculopathy. This typically presents with neurologic symptoms such as paresthesia, sensory loss, decreased strength, and/or diminished reflexes.^16^ Spondylosis, or the arthritic change of the spinal discs and facet joints, presents as back pain with radiation to the buttock and/or thigh along with neurologic deficits in the L5 – S1 spinal nerve root distribution.^15^ Lumbar spinal stenosis is the narrowing of the spinal canal, which presents as low back pain relieved by rest. Neurologic exam may be normal or include decreased muscle strength or sensation.^15^ Fracture can occur with significant trauma or as a result of a vertebral compression. Physical exam may show focal tenderness on palpation and history may include risk factors such as glucocorticoid use, increased age, and osteoporosis.^15^

Finally, non‐orthopedic causes of posterior hip and low back pain include malignancy and AAA. Due to this patient's age, malignancy must be a consideration. Patient history may reveal previous malignancy, unexplained weight loss, and/or constitutional symptoms. On physical exam, we would expect focal tenderness to palpation.^15^ Additionally, given the patient's significant cardiac and vascular history, AAA cannot be ruled out without imaging. Unruptured AAA can present as abdominal pain radiating to the flank, back, or groin.^4^, ^11^

Given the acute nature of the patient's pain, the HPI, and the findings on physical exam, the leading diagnosis is posterior hip and low back pain likely due to muscle strain and resultant inflammation. Due to the patient's history of bilateral THA, orthopedic complications such as prosthetic wear, aseptic loosening, periprosthetic fracture, and leg length discrepancy should not be ruled out without further imaging. Both malignancy and AAA are “Do Not Miss” differentials and must also be ruled out with imaging.

### 2.4 Investigations

**FIGURE 1:**
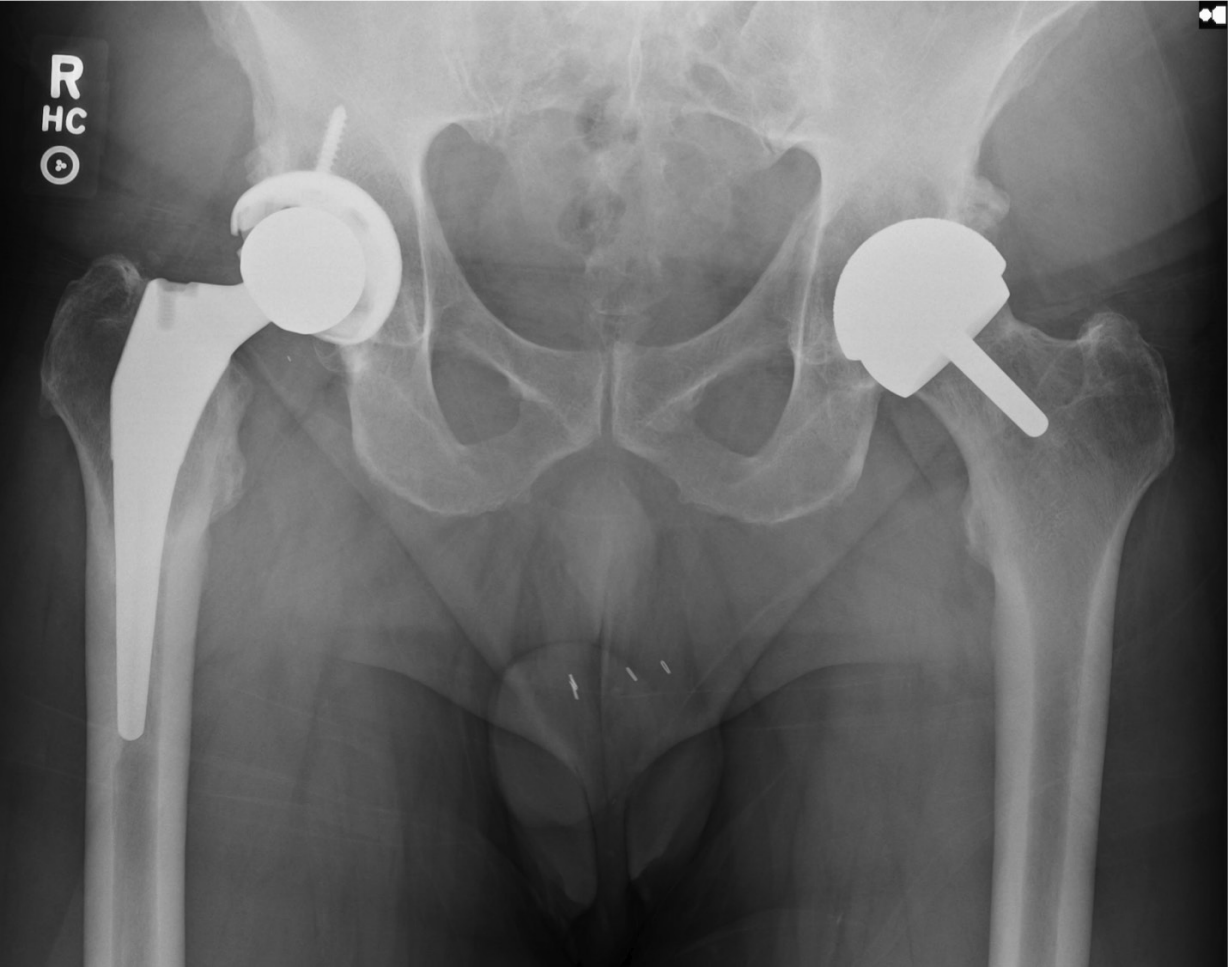
Anterior–posterior radiograph of bilateral total hip arthroplasty.

**FIGURE 2:**
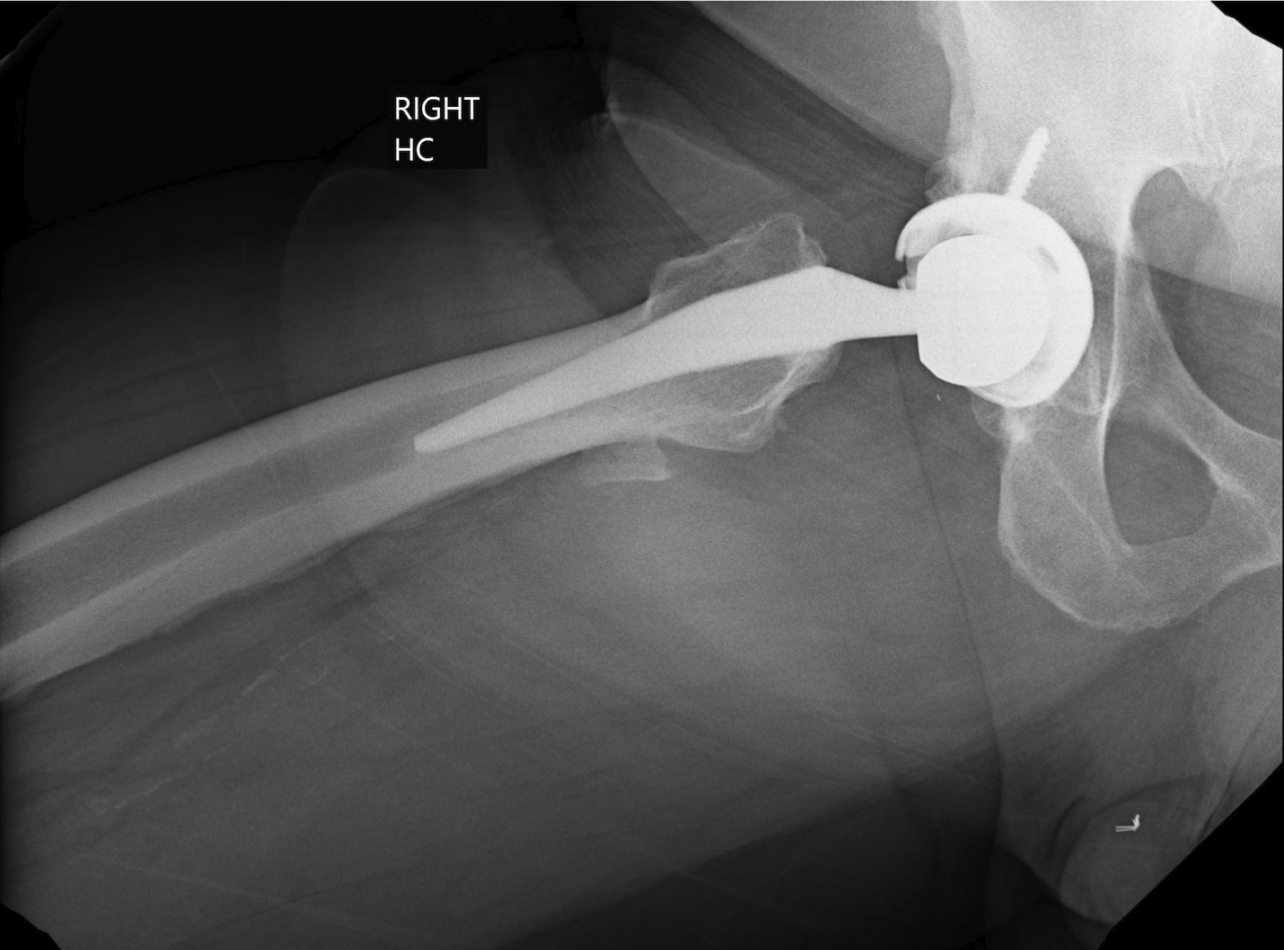
Lateral radiograph of right total hip arthroplasty.

**FIGURE 3:**
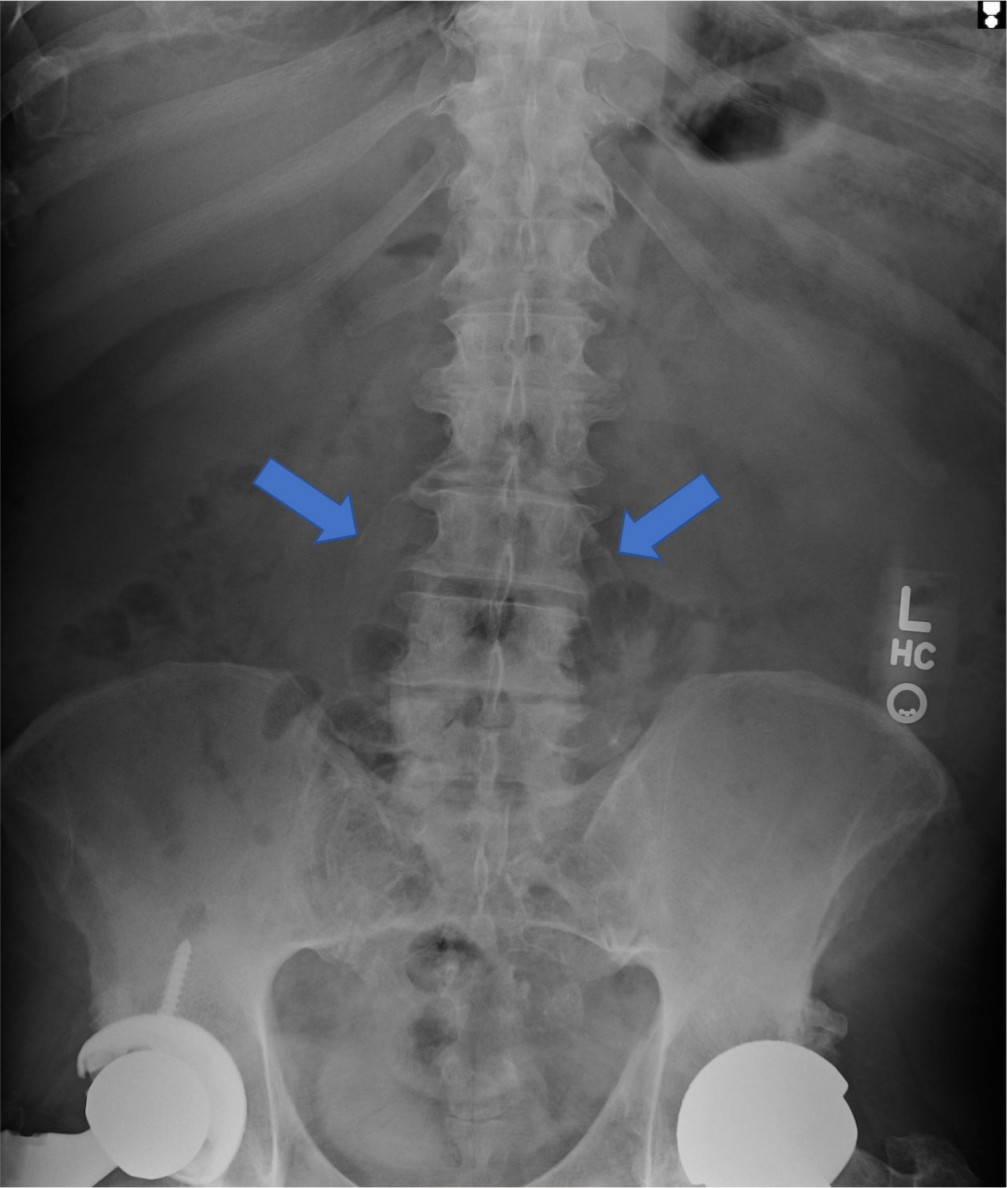
Anterior–posterior radiograph of lumbar spine demonstrating calcified outline of abdominal aortic aneurysms (blue arrows).

**FIGURE 4:**
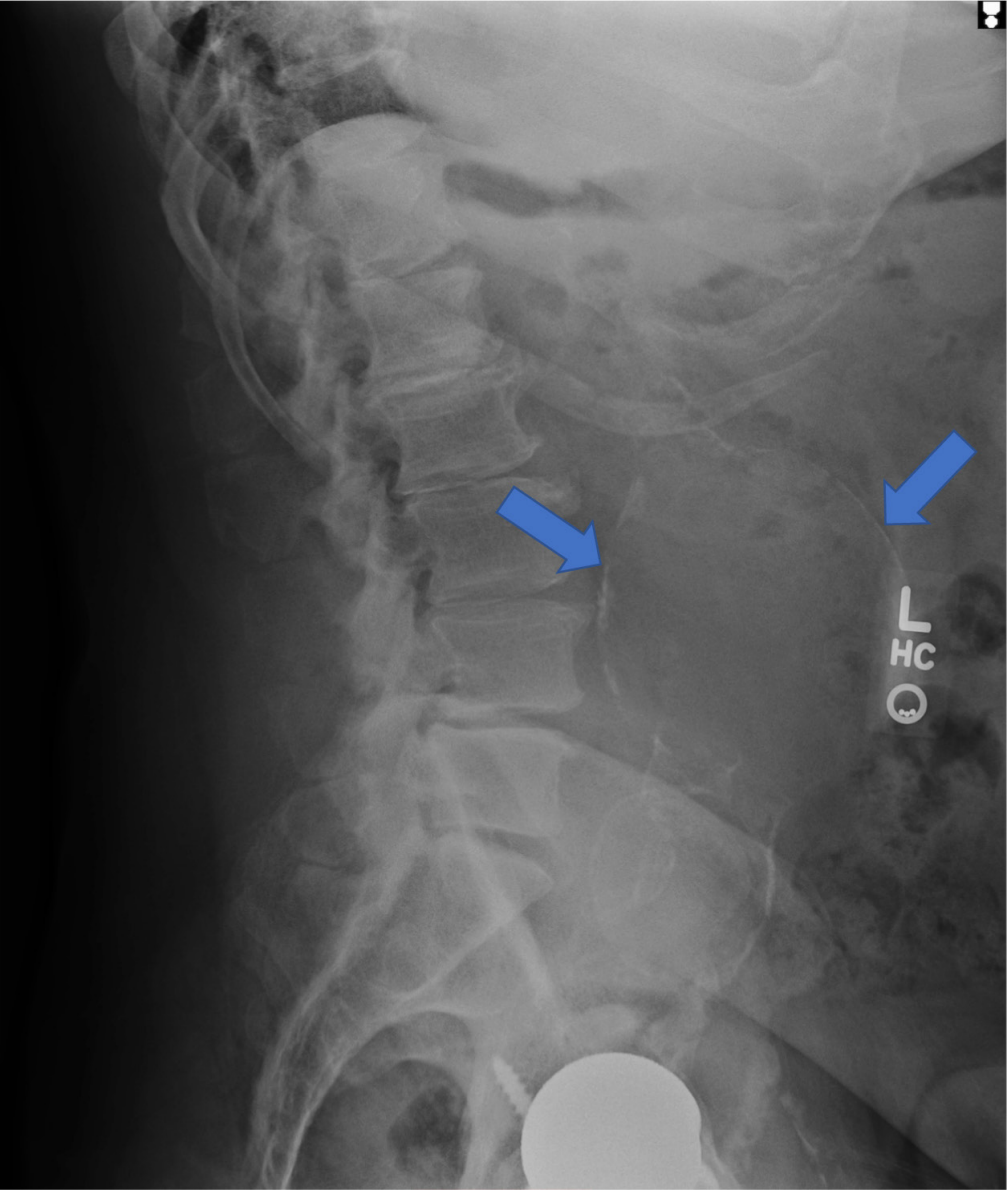
Lateral radiograph of lumbar spine demonstrating calcified outline of abdominal aortic aneurysms (blue arrows).

Anterior–posterior (AP) and lateral x‐rays of the right hip (Figures 1 and 2) and lumbar spine (Figures 3 and 4) were obtained. The radiographs were unremarkable for any bony abnormalities, fractures, or dislocations. On the AP and lateral views of the lumbar spine (Figures 3 and 4), a calcified outline of the aorta in the anterior aspect of the lumbar spine measuring approximately 10‐cm in diameter was observed. The imaging studies were highly suggestive of a large, unruptured AAA.

### 2.5 Outcome and follow‐up

The patient was transported via private auto to the emergency department at the University of Connecticut John Dempsey Hospital for immediate evaluation of his AAA. The patient was then recommended to follow‐up with a hip specialist for further evaluation after his AAA was treated.

Verbal follow‐up included a call from the emergency department provider to the orthopedic urgent care to confirm the diagnosis of AAA and provide patient outcome.

## 3 DISCUSSION

Our case report demonstrates an incidental finding of a large, unruptured AAA during a workup for a chief concern of posterior right hip and low back pain. Unruptured AAA is typically asymptomatic. Occasionally, unruptured AAA can become symptomatic and mimic MSK pain in the flank, back, and/or groin.^4^, ^11^ In the orthopedic clinic, an AAA is not high on the differential for a patient presenting with posterior hip and low back pain. In our case, the patient presented to an orthopedic‐only urgent care. In this setting, any non‐MSK pathology is usually not considered. However, there is crucial information in the patient's history and physical exam that should steer the provider away from an MSK etiology and toward the diagnosis of an unruptured AAA.

With a chief concern of posterior hip and low back pain in a patient with a history of bilateral THA, the initial assumption would be a muscle strain or a complication related to the patient's prostheses. There are aspects of the patient's physical exam that align with this orthopedic etiology. First, the patient ambulated with a painful right leg and their gait was antalgic. Additionally, there was reproducible tenderness on the posterior aspect of the right lumbosacral junction. Reproducible pain is a commonly known sign of MSK pathology. However, this physical exam finding is not specific for MSK etiologies and cannot rule out other causes.

Contrarily, there were important aspects in the HPI and physical exam findings that point away from MSK pathology. First, the patient denied any trauma, injury, or falls. Mechanism of injury (MOI) is a vital component of orthopedic assessment. With no known MOI, certain injuries such as muscle strain or periprosthetic fractures are lower on the differential. However, non‐traumatic causes such as infection and osteolysis must still be considered. An important note from the patient's physical exam was the finding that range of motion was fully intact with no discomfort. Again, this points away from MSK pathology.

The patient's past medical history is, perhaps, the most important consideration that would direct a provider toward the diagnosis of an AAA. The patient's history is significant for hypertension, hyperlipidemia, CAD, T2DM, and PAD; all of which are high risk factors for the development of an AAA. Furthermore, the patient is >65 years old and male. Both are also risk factors for an AAA.^3^, ^4^, ^5^, ^6^, ^7^, ^8^ The patient's significant cardiac and vascular history, in conjunction with the HPI and negative findings on orthopedic physical exam, should logically lead a provider toward a non‐orthopedic etiology and raise suspicion for an AAA.

Hip and back pain is an atypical, but known, presentation for an AAA. This clinical scenario is similar to other cases described in the literature. Smith et al. reported a 66‐year‐old male patient with an 8‐month history of progressive left hip pain who was incidentally found to have an unruptured AAA.^17^ Baskaran et al. described a 58‐year‐old patient with a 6‐month history of progressively worsening left hip pain associated with unintentional weight loss of 38 kg and tender bilateral testicular swelling.^18^ These two cases demonstrated chronic hip pain, unlike the patient in our report who presented with a 3‐day history of pain. A complaint of chronic pain is more typical of a symptomatic unruptured AAA, due to the insidious growth of the dilatation. Our case is unique in this regard. Furthermore, low back pain has been a well‐documented presentation of an unruptured AAA in several case reports.^19^, ^20^, ^21^, ^22^, ^23^, ^24^, ^25^

Our case report emphasizes the necessity of extracting a thorough HPI and performing a holistic physical exam in the orthopedic clinic. Orthopedic providers should consider an unruptured AAA as an atypical differential diagnosis when a patient presents with hip and/or low back pain. There should be a particularly high index of suspicion for an AAA in the context of a significant cardiac and vascular history, associated risk factors (i.e., smoking, T2DM, atherosclerosis, hypertension, etc.), and negative orthopedic findings on physical exam. Awareness and education of this presentation is crucial for avoiding a missed diagnosis. A missed diagnosis of an unruptured AAA may put the patient at risk of catastrophic rupture and subsequent death.

Orthopedic surgery is a field that tends to have a myopic approach to patient care. With increasingly advanced subspecialties, orthopedic surgeons are highly skilled in their respective disciplines. Inadvertently, differential diagnoses that are typically outside the field of practice may not be considered. However, it is important for orthopedic providers to be well rounded in all aspects of patient care and perform holistic investigations when assessing a patient.

This case report has limited generalizability, as it discusses a single case. However, it highlights important aspects of patient care and can be used as a teaching tool to advocate for holistic practices in the orthopedic clinic.

## 4 CONCLUSION

In conclusion, an unruptured AAA may be an atypical cause of posterior hip and low back pain. Orthopedic providers should include an unruptured AAA on the differential for a patient presenting with these concerns, particularly in the context of significant cardiac and vascular history, significant risk factors, and negative orthopedic findings on physical exam. Additionally, orthopedic providers should focus on a holistic approach to patient care, considering all aspects of the history of present illness, past medical history, family history, social history, and physical exam when exploring differential diagnoses.

## AUTHOR CONTRIBUTIONS

**Aaron Marcel:** Conceptualization; formal analysis; investigation; methodology; validation; visualization; writing – original draft; writing – review and editing. **John Jovan:** Investigation; methodology; writing – original draft; writing – review and editing. **Karen Myrick:** Conceptualization; investigation; methodology; supervision; writing – original draft; writing – review and editing.

## FUNDING INFORMATION

No funding source supported the production of this study.

## CONFLICT OF INTEREST STATEMENT

All authors have no conflicts of interest to declare.

## CONSENT

Written informed consent was obtained from the patient to publish this report in accordance with the journal's patient consent policy.

## Data Availability

Data are not available due to ethical restrictions.
